# Prevalence and oral hygiene–related correlates of self-reported dentin hypersensitivity among Egyptian adults: a cross-sectional study

**DOI:** 10.1186/s12903-026-09900-4

**Published:** 2026-10-05

**Authors:** Dalia Ghalwash, Ahmed Hamdy, Shehad Wael, Al-Hassan Diab, Ahmed Ammar

**Affiliations:** Department of Oral Medicine and Periodontology, Faculty of Dentistry, The British University in Egypt, El Sherouk City, Cairo, 11837 Egypt

**Keywords:** Dentin hypersensitivity, Self-reported dentin hypersensitivity prevalence, Gingival recession, Cross-sectional study, Oral hygiene, Egypt

## Abstract

**Background:**

Dentin hypersensitivity (DH) is a prevalent clinical condition characterized by pain arising from exposed dentin in response to external stimuli. Despite its clinical relevance, epidemiological data from Egypt and the Middle East remain limited. This study aimed to assess the prevalence, symptom characteristics, and oral hygiene-related correlates of self-reported dentin hypersensitivity among Egyptians adults.

**Methods:**

A cross-sectional questionnaire-based study was conducted among 370 Egyptian adults recruited from multiple governorates between January and August 2025. Data was collected using a structured questionnaire that underwent expert review, pilot testing, and reliability assessment. Information regarding self-reported dentin hypersensitivity, triggering stimuli, oral hygiene habits, behavioral factors, and sociodemographic characteristics was obtained. Multivariable logistic regression analyses were performed to explore factors associated with self-reported gingival recession and frequent or persistent dentin hypersensitivity symptoms.

**Results:**

Self-reported dentin hypersensitivity was reported by 86 participants, yielding an overall prevalence of 23.2% (95% CI: 19.2–27.8%). The most common trigger was cold stimuli, and the most common symptom pattern was intermittent discomfort. Most participants reporting hypersensitivity were younger adults, although no significant association with sex was observed. Self-reported gingival recession was reported by 27.9% of participants with dentin hypersensitivity. Multivariable regression analyses did not identify significant independent predictors of self-reported gingival recession or frequent/persistent hypersensitivity symptoms.

**Conclusion:**

Self-reported dentin hypersensitivity affected nearly one-quarter of surveyed Egyptian adults, with cold-triggered symptoms predominating. The absence of strong independent predictors supports the multifactorial nature of dentin hypersensitivity. These findings provide region-specific epidemiological data and highlight the need for future studies incorporating standardized clinical assessment to validate self-reported symptoms and further investigate associated factors.

**Supplementary Information:**

The online version contains supplementary material available at https://doi.org/10.1186/s12903-026-09900-4.

## Introduction

Dentin hypersensitivity (DH) is a common clinical condition characterized by a short, sharp pain arising from exposed dentin in response to thermal, evaporative, tactile, osmotic, or chemical stimuli that cannot be attributed to another dental defect or pathology [1, 2]. Even though it is frequently encountered in dental practice, DH remains challenging to diagnose and manage because symptoms are often episodic, vary considerably in intensity, and may overlap with other painful dental problems, leading to underrecognition and delayed management [3, 4].

The prevalence of DH varies substantially across populations and study settings. A systematic review and meta-analysis reported pooled prevalence estimates generally ranging between 20% and 35%, although considerable heterogeneity exists due to differences in study design, diagnostic criteria, assessment methods, and population characteristics [5]. Studies conducted in dental clinics often report higher prevalence rates than community-based investigations, likely reflecting treatment-seeking behavior and selection bias [6, 7]. Despite the increasing international literature, epidemiological data from Egypt and the wider Middle East and North Africa region remain limited, highlighting the need for population-specific investigations [8].

The most widely accepted explanation for DH is Brännström’s hydrodynamic theory, which proposes that external stimuli induce fluid movement within exposed dentinal tubules, activating mechanosensitive nerve endings in the pulp–dentin complex and producing pain [2, 4]. Dentin exposure may occur as a result of gingival recession, tooth wear, erosion, abrasion, attrition, or non-carious cervical lesions [9, 10]. Although several behavioral and environmental factors, including toothbrushing habits, smoking, and dietary acid exposure, have been implicated, current evidence suggests that DH is a multifactorial condition resulting from the interaction of biological, behavioral, and environmental influences rather than a single dominant risk factor [11, 12].

Beyond its biological basis, DH may have important functional and psychosocial consequences. Previous studies have demonstrated that hypersensitivity can negatively affect food and beverage choices, oral hygiene practices, daily comfort, and oral health-related quality of life [13–15]. Pain-related avoidance behaviors may also contribute to changes in toothbrushing habits and oral hygiene maintenance [16]. Nevertheless, many affected individuals rely on self-management strategies rather than seeking professional care, potentially delaying diagnosis and treatment [17].

Given the limited epidemiological evidence from Egypt and neighboring countries, the present cross-sectional study aimed to determine the prevalence of self-reported dentin hypersensitivity among Egyptian adults and to characterize its symptom patterns, triggering factors, and oral hygiene–related behaviors. Additionally, factors associated with self-reported gingival recession and severe hypersensitivity symptoms among affected individuals were explored. By providing population-specific data from an underrepresented region, this study contributes to the evidence base needed to support preventive strategies, patient education, and evidence-based management of dentin hypersensitivity.

## Methods

### Study design and setting

This cross-sectional questionnaire-based study was conducted to investigate the prevalence, characteristics, and oral hygiene–related correlates of self-reported dentin hypersensitivity (DH) among Egyptian adults. The Research Ethics Committee of the British University of Egypt’s Faculty of Dentistry approved the study procedure, with approval No. 23–070, which was carried out in compliance with the principles of the Declaration of Helsinki [18]. All participants provided informed consent prior to enrollment. Questionnaires were completed anonymously, and no personally identifiable information was collected.

Participants were recruited between January and August 2025 from Cairo, Giza, Qalyubia, and Sharkia governorates through dental clinics and supervised community outreach activities conducted in clinical settings. Recruitment was performed directly by trained investigators and did not involve social media, open online invitations, or anonymous public participation. The study is reported in accordance with the Strengthening the Reporting of Observational Studies in Epidemiology (STROBE) guidelines for cross-sectional studies [19].

### Sample size calculation

The sample size was calculated based on the primary outcome of self-reported dentin hypersensitivity prevalence. The formula for prevalence studies, n = (Z^2⋅p⋅(1-p)/d^2), was used to determine the minimum necessary sample size using data from a comprehensive review that reported a 30% prevalence among adults [5], a 95% confidence level (Z = 1.96), and a 5% margin of error (d = 0.05).

Consequently, 323 people made up the necessary sample. A final objective of about 370 participants was reached when the sample size was expanded by 15% to account for any non-response or incomplete questionnaires. This sample size guarantees sufficient accuracy in determining the dentin hypersensitivity prevalence among Egyptian adults.

### Participants and sampling

**Fig. 1:**
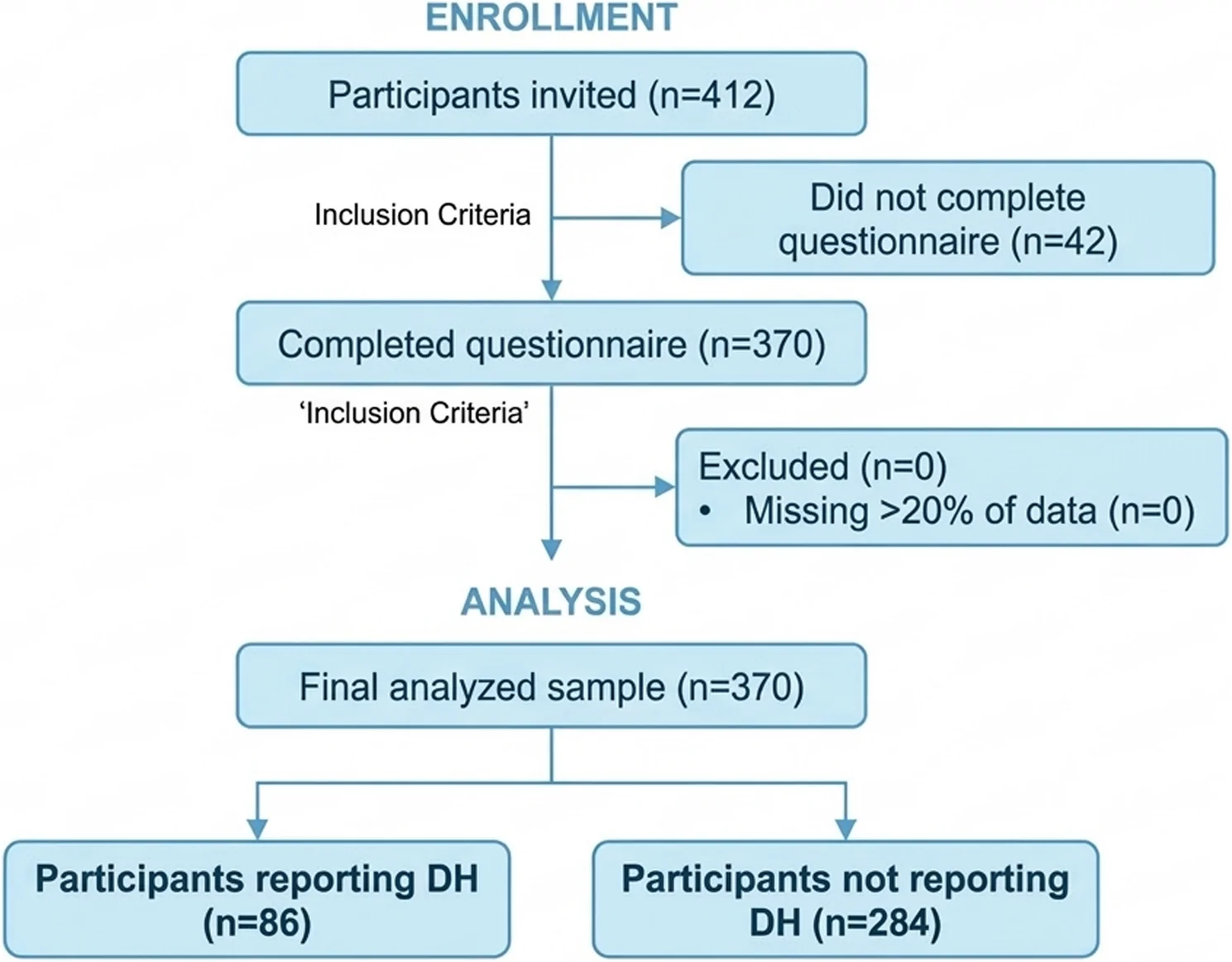
Participants' Flow through the study (STROBE Diagram), n = number

Adult residents aged ≥ 18 years, who were able to provide informed consent and complete the questionnaire, were invited to participate. Participants were recruited using a convenience sampling approach from dental clinics and supervised community outreach activities conducted in clinical settings across multiple governorates in Egypt. Recruitment was carried out directly by trained investigators, who screened participants for eligibility before questionnaire administration. Individuals with conditions that could impair reliable self-reporting, including significant cognitive impairment or severe oral pain conditions such as acute pulpitis, dental abscesses, or extensive untreated dental caries, were excluded. Recruitment did not involve social media, open online invitations, or anonymous public participation. Although individuals aged 18 years and older were eligible for participation, no respondents aged 18–21 years were included in the final dataset; therefore, the youngest age category reported in the analyses was 22–29 years. No quota sampling, stratification, or intentional balancing by sex was applied; the nearly equal distribution of male and female participants occurred naturally during the recruitment process. Participant recruitment and inclusion are summarized in Fig. 1.

### Questionnaire development and validation

Data were collected using a structured self-administered questionnaire designed to assess the prevalence, characteristics, and oral hygiene–related factors associated with self-reported dentin hypersensitivity. Questionnaire development was informed by previously published epidemiological studies and methodological recommendations related to dentin hypersensitivity assessment [2, 13, 20].

The questionnaire was adapted from previously published studies [13, 20], and translated into Arabic using forward–backward translation to ensure linguistic accuracy and cultural appropriateness.

### Content validity

Content validity was assessed by three independent external academic experts in periodontology and dental medicine, none of whom were members of the authorship team. The experts evaluated the questionnaire for relevance, clarity, comprehensiveness, and cultural suitability. Minor modifications were implemented based on their recommendations.

### Pilot testing

The preliminary instrument was pilot tested on 30 adult participants representative of the target population to assess clarity, comprehension, and completion time. Participant feedback resulted in minor refinements to item sequencing and response options. Data obtained during the pilot phase were not included in the final analysis.

### Questionnaire structure

The final questionnaire consisted of six sections:

1. Informed consent
2. Sociodemographic characteristics.
3. Lifestyle and medical history.
4. Dentin hypersensitivity profile.
5. Oral hygiene practices.
6. Additional oral health factors.aa

Self-reported DH presence, pain characteristics (sharp, dull, throbbing), triggering stimuli (thermal, tactile, osmotic, chemical), frequency of symptoms, anatomical location, and treatment-seeking behavior were all evaluated in the dentin hypersensitivity domain.

Oral hygiene–related variables included toothbrushing frequency, brushing technique, brushing force, toothbrush bristle hardness, toothpaste type, smoking status, and awareness of bruxism.

Gingival recession was assessed by self-report and was not clinically verified. Likewise, all dentin hypersensitivity data were based on participant-reported symptoms without clinical examination or provocation testing.

### Data collection procedure

The questionnaire was administered electronically using Google Forms. Participation was anonymous, and no personally identifiable information was collected. Responses were automatically coded for statistical analysis. Questionnaires with more than 20% missing responses were excluded from analysis.

### Statistical analysis

Data was analyzed using IBM SPSS Statistics version 25.0 (IBM Corp., Armonk, NY, USA). Categorical variables were summarized as frequencies and percentages. Associations between categorical variables were assessed using the Chi-square test or Fisher’s exact test, as appropriate. Comparisons were conducted primarily between participants with and without self-reported dentin hypersensitivity (DH).

To identify factors independently associated with self-reported gingival recession and severe dentin hypersensitivity, multivariable binary logistic regression analyses were performed. Severe DH was defined as frequent or persistent hypersensitivity symptoms, consistent with symptom-based classifications commonly used in epidemiological studies of dentin hypersensitivity [13, 20]. Predictor variables were selected a priori based on biological plausibility and evidence from previous literature. Owing to the relatively small number of participants reporting gingival recession, the corresponding regression model was limited to three clinically relevant predictors to reduce the risk of overfitting.

Adjusted odds ratios (aORs) with 95% confidence intervals (95% CIs) were reported. Multicollinearity among independent variables was assessed using variance inflation factors (VIFs). Model calibration was evaluated using the Hosmer–Lemeshow goodness-of-fit test.

Cases with missing responses were excluded from the relevant analyses (complete-case analysis). All statistical tests were two-tailed, and a p-value < 0.05 was considered statistically significant.

## Results

### Study sample and prevalence of dentin hypersensitivity

A total of 370 adults participated in this cross-sectional survey, including 185 females and 185 males. DH was reported by 86 participants, yielding an overall prevalence of 23.2% (95% CI 19.2% to 27.8%) (Table 1). The prevalence was comparable between females (24.3%) and males (22.2%), with an odds ratio for females vs. males of 1.13, 95% CI: 0.70 to 1.83, *p* = 0.623.

**Table 1:** Prevalence of self-reported dentin hypersensitivity in the study population (*n* = 370)

Outcome	*n*	%
Self-reported DH	86	23.2
No self-reported DH	284	76.8

Subsequent analyses of symptom characteristics, triggering factors, oral hygiene practices, treatment-seeking behavior, and regression models were restricted to participants reporting self-reported dentin hypersensitivity (*n* = 86).

### Reliability assessment

The questionnaire demonstrated good internal consistency, with an overall Cronbach’s alpha coefficient of 0.82. Subscale reliability coefficients were 0.79 for symptom characteristics, 0.85 for triggering stimuli, and 0.76 for oral hygiene practices. Test–retest reliability was assessed in a subgroup of 25 participants who completed the questionnaire twice within a two-week interval. The intraclass correlation coefficient was 0.87, indicating excellent temporal stability.

### Sociodemographic characteristics

Among participants reporting self-reported DH, 52.3% were aged 22–29 years, and 27.9% were aged 30–39 years. Females represented 52.3% of affected participants. Most participants were non-smokers (75.6%) (Table 2).

**Table 2:** Sociodemographic characteristics of participants reporting self-reported dentin hypersensitivity (*n* = 86)

Variables		Dentin hypersensitivity	
		n	%
Age	22–29 years	45	52.3%
	30–39 years	24	27.9%
	40–49 years	10	11.6%
	50–59 years	5	5.8%
	60 years and more	2	2.3%
Gender	Female	45	52.3%
	Male	41	47.7%
Smoking	No	65	75.6%
	Yes (≤ 20 cigarettes /day)	17	19.8%
	Yes (≥ 20 cigarettes /day)	3	3.5%
	Other	1	1.2%

### Characteristics of dentin hypersensitivity

Among participants reporting DH, dull pain was the most frequently reported symptom (54.7%), followed by sharp pain (34.9%) and throbbing pain (10.5%). Cold stimuli represented the most commonly reported trigger (37.2%), although multiple triggers were frequently reported. Most participants described symptoms as occasional (60.5%), whereas 39.5% reported frequent or persistent symptoms. Multiple tooth locations were affected in 38.4% of participants (Table 3).

**Table 3:** Clinical characteristics of self-reported dentin hypersensitivity (*n* = 86)

Type of DH	Sharp	30	34.9%
Stimuli that trigger DH	Dull	47	54.7%
	Throbbing	9	10.5%
	Hot	2	2.3%
	Cold	32	37.2%
	Sweet	3	3.5%
	Sour	0	0%
	Citrus fruit	3	3.5%
	Mouthwash	0	0%
	Toothbrush	4	4.7%
	Chewing	5	5.8%
	Other and Combinations	37	43%
Frequency of DH	Occasionally	52	60.5%
	Often	31	36%
	Constant	3	3.5%
Location of teeth affected	Upper right jaw	12	14%
	Lower right jaw	10	11.6%
	Upper left jaw	10	11.6%
	Lower left jaw	11	12.8%
	Upper anterior	1	1.2%
	Lower anterior	9	10.5%
	Combinations	33	38.4%

### Oral hygiene practices and self-reported gingival recession

Only 29 participants (33.7%) reported previously seeking professional treatment for DH. Medium-bristle toothbrushes were most commonly used (59.3%), and approximately half of the participants brushed once daily (48.8%). Excessive brushing force was reported by 23.3% of respondents. Fluoride toothpaste was used by 65.1% of participants, whereas 31.4% reported using desensitizing toothpaste. Self-reported gingival recession was reported by 24 participants (27.9%) (Table 4).

**Table 4:** Oral hygiene practices and self-reported gingival recession among participants reporting self-reported dentin hypersensitivity (*n* = 86)

Sought treatment for DH	Yes	29	33.7%
	No	57	66.3%
Bristles hardness of toothbrushes used	Soft	32	37.2%
	Medium	51	59.3%
	Hard	3	3.5%
Daily frequency of toothbrushing	None	4	4.7%
	Once	42	48.8%
	Twice	32	37.2%
	3 or more times	8	9.3%
Toothbrushing with excessive force	Yes	20	23.3%
	No	66	76.7%
Toothbrushing techniques	Horizontal	23	26.7%
	Horizontal-Vertical	14	16.3%
	Vertical	8	9.3%
	Circular	33	38.4%
	Random	8	9.3%
Choice of toothpaste	De-sensitizing	27	31.4%
	Fluoride	56	65.1%
	Other	3	3.5%
Awareness of bruxism or grinding of teeth	No	45	52.3%
	Yes	41	47.7%
Gingival recession	None	62	72.1%
	Upper right jaw	3	3.5%
	Lower right jaw	4	4.7%
	Upper left jaw	2	2.3%
	Lower left jaw	2	2.3%
	Upper anterior	0	0%
	Lower anterior	3	3.5%
	Combinations	10	11.6%

### Gingival recession model

Self-reported gingival recession was reported by 24 participants (27.9%). In the multivariable logistic regression model, toothbrushing frequency demonstrated a borderline inverse association with gingival recession (aOR = 0.48, 95% CI: 0.22–1.05, *p* = 0.066). Age category, smoking status, and excessive brushing force were not significantly associated with self-reported gingival recession (Table 5).

**Table 5:** Multivariable logistic regression for gingival recession (present vs. none)

Predictor	aOR	95% CI (Lower)	95% CI (Upper)	*p*-value
Age category (per category increase)	1.05	0.64	1.73	0.845
Smoking (smoker vs. non-smoker)	0.75	0.23	2.46	0.638
Excessive brushing force (yes vs. no)	0.98	0.31	3.13	0.978
Toothbrushing frequency (per category increase)	0.48	0.22	1.05	0.066

The model demonstrated acceptable calibration according to the Hosmer–Lemeshow goodness-of-fit test (χ² = 10.36, df = 8, *p* = 0.241).

### Severe dentin hypersensitivity model

Frequent or persistent symptoms, classified as severe DH, were reported by 34 participants (39.5%). In multivariable logistic regression analysis, age category, smoking status, and excessive brushing force were not independently associated with severe DH (Table 6).

**Table 6:** Conservative multivariable logistic regression model for severe DH

Predictor	aOR	95% CI (Lower)	95% CI (Upper)	*p*-value
Age	1.02	0.66	1.58	0.918
smoker	1.54	0.56	4.22	0.401
Excessive force	1.33	0.47	3.73	0.590

The model demonstrated acceptable calibration (Hosmer–Lemeshow χ² = 4.78, df = 4, *p* = 0.311).

Additional analyses showed no significant association between self-reported gingival recession and DH severity (OR = 1.08, 95% CI: 0.40–2.90; Fisher’s exact *p* = 1.000). Similarly, neither toothbrushing technique (χ² = 8.39, *p* = 0.078) nor toothpaste type (χ² = 2.50, *p* = 0.287) was significantly associated with DH severity.

## Discussion

The present cross-sectional study investigated the prevalence, characteristics, and oral hygiene–related correlates of self-reported DH among Egyptian adults. Approximately one-quarter of the surveyed participants reported experiencing DH, indicating that dentin sensitivity represents a common oral health complaint within this population. While several oral hygiene–related factors and symptom characteristics were identified among affected individuals, multivariable analyses did not reveal strong independent predictors of either self-reported gingival recession or frequent/persistent hypersensitivity symptoms. Collectively, these findings support the contemporary view that dentin hypersensitivity is a multifactorial condition influenced by complex interactions among biological, behavioral, environmental, and patient-related factors rather than a single dominant determinant [1, 4, 8, 12].

The prevalence of self-reported dentin hypersensitivity in the present study (23.2%) falls within the range reported internationally. A systematic review and meta-analysis by Favaro Zeola et al. reported pooled prevalence estimates generally ranging between 20% and 35%, although substantial heterogeneity was observed due to differences in study design, diagnostic criteria, and population characteristics [5]. More recent epidemiological investigations from Turkey and other populations have similarly demonstrated considerable variability in prevalence estimates, reflecting differences in sampling methods, participant demographics, and diagnostic approaches [7, 11]. Similar prevalence figures have been reported in community-based studies from Asia and Europe, whereas higher estimates are often observed among dental clinic attendees, likely reflecting treatment-seeking behavior and selection bias [4, 6]. Consistent with previous studies, cold stimuli represented the most commonly reported individual trigger of hypersensitivity symptoms. These observations suggest that the burden of self-reported DH among Egyptian adults is broadly comparable to that reported in other populations despite differences in cultural, behavioral, and healthcare-related factors.

A major contributor to the variability in reported prevalence estimates is the distinction between clinically confirmed dentin hypersensitivity and self-reported symptoms. Previous investigations have demonstrated that patient-reported sensitivity frequently exceeds clinically confirmed cases, emphasizing the diagnostic challenges associated with epidemiological studies of DH and the importance of standardized assessment protocols [4, 8, 20]. Consequently, the prevalence reported in the present study should be interpreted as reflecting the burden of self-reported DH symptoms rather than clinically diagnosed dentin hypersensitivity. Nevertheless, patient-reported symptoms remain clinically relevant because they reflect perceived discomfort and may influence oral hygiene practices, dietary habits, and healthcare-seeking behavior [1, 13].

The predominance of younger adults among participants reporting hypersensitivity is also consistent with previous epidemiological investigations. Dentin hypersensitivity is generally most prevalent during early and middle adulthood, after which dentinal sclerosis and progressive tubule occlusion may reduce dentin permeability and symptom expression [2, 4]. Similar age-related patterns have been reported in studies conducted in the United States and Turkey [6, 7, 11]. However, unlike some studies that reported associations with sex, smoking, or oral hygiene habits, the present study did not identify significant independent predictors after adjustment, further supporting the multifactorial nature of self-reported dentin hypersensitivity. Although females reported DH slightly more frequently than males, the difference was not statistically significant. Similar findings have been reported in several population-based studies, suggesting that observed sex differences may be influenced more by behavioral, psychosocial, and health-seeking factors than by biological sex alone [1, 5].

Cold stimuli were among the most commonly reported triggers of hypersensitivity, while symptoms were reported predominantly on an occasional basis. These findings are generally consistent with the hydrodynamic theory proposed by Brännström, which remains the most widely accepted explanation for dentin hypersensitivity. According to this theory, thermal, evaporative, tactile, or osmotic stimuli induce rapid fluid movement within exposed dentinal tubules, activating mechanosensitive nerve endings within the pulp–dentin complex and generating pain [1, 3, 4, 20]. Although cold sensitivity was commonly reported and is consistent with the classical clinical presentation of dentin hypersensitivity, dull pain was the most frequently reported pain quality in the present study. This differs from the widely accepted definition of DH as a short, sharp pain elicited by external stimuli. The discrepancy likely reflects the inherent limitations of questionnaire-based self-report, whereby participants may have described symptoms arising from other dental conditions or interpreted pain descriptors differently despite the applied exclusion criteria. Consequently, self-reported DH should not be considered equivalent to a clinical diagnosis, and the findings should be interpreted accordingly.

Interestingly, the absence of significant associations between self-reported gingival recession and frequent/persistent hypersensitivity symptoms in multivariable analyses highlights the biological complexity of DH. Although gingival recession is frequently considered a major predisposing factor because it exposes root dentin, exposure alone does not necessarily result in symptoms. Experimental and clinical studies have demonstrated that the density, diameter, and patency of dentinal tubules are critical determinants of sensitivity. Exposed dentin may remain asymptomatic when dentinal tubules become partially occluded by smear layers, mineral deposition, or other natural protective mechanisms [2, 12, 21]. Consequently, considerable interindividual variation may exist between the extent of dentin exposure and the severity of perceived symptoms.

The predominance of intermittent rather than persistent pain observed in the present study further supports the stimulus-dependent nature of DH. Unlike pulpal pain, which may occur spontaneously and continuously, hypersensitivity episodes are generally elicited only in response to specific external stimuli. This characteristic may partly explain why many individuals do not seek professional care despite experiencing recurrent symptoms. Indeed, only a minority of affected participants reported consulting a dentist regarding their hypersensitivity. Similar observations have been reported elsewhere and suggest that many individuals perceive DH as a manageable inconvenience rather than a condition requiring professional intervention [2, 8, 17].

The behavioral findings of this study also warrant consideration. Approximately one-quarter of participants with DH reported using excessive brushing force, yet this variable did not emerge as an independent predictor of either frequent/persistent symptoms or self-reported gingival recession. This observation aligns with current evidence suggesting that dentin exposure and hypersensitivity typically result from the cumulative effects of multiple factors rather than a single behavioral habit. Mechanical abrasion associated with toothbrushing, erosive dietary challenges, attrition, corrosion, and non-carious cervical lesions may collectively contribute to dentin exposure and hypersensitivity development [9, 10, 22, 23]. Therefore, the limited predictive value of isolated behavioral variables observed in the present study should not be interpreted as evidence that such factors are unimportant, but rather that their effects are likely mediated through complex interactions that could not be fully captured within a cross-sectional design.

Approximately one-quarter of participants with self-reported DH also reported gingival recession. Although recession is widely recognized as an important risk factor for root dentin exposure, its development is influenced by numerous factors, including periodontal phenotype, gingival thickness, anatomical characteristics, cumulative inflammatory burden, and oral hygiene practices [24]. The multifactorial nature of recession may partly explain why no single variable emerged as a strong independent predictor in the present analysis. Furthermore, because gingival recession was self-reported rather than clinically assessed, some degree of misclassification may have reduced the ability to detect significant associations.

The findings also highlight the potential functional and psychosocial impact of hypersensitivity symptoms. Nearly 40% of affected participants reported frequent or persistent symptoms, suggesting that a substantial subset experiences ongoing discomfort. Previous investigations have demonstrated that dentin hypersensitivity may negatively affect eating habits, beverage consumption, oral hygiene practices, daily comfort, social interactions, and overall oral health-related quality of life [13–15, 25]. Pain-related avoidance behaviors may further compromise oral hygiene practices, potentially contributing to a cycle of symptom persistence and oral health deterioration [16]. Similar impacts have been reported in Egyptian populations affected by chronic oral conditions, including recurrent aphthous ulcers, recurrent herpes labialis, and burning mouth syndrome, where symptom burden was associated with measurable psychosocial consequences and reduced quality of life [26–28]. Although quality-of-life measures were not assessed in the present study, the frequency and persistence of symptoms reported by participants suggest that the burden of DH may extend beyond transient discomfort and influence everyday functioning and well-being.

Several clinical implications emerge from these findings. First, self-reported dentin hypersensitivity appears to be a common complaint among Egyptian adults and should not be overlooked during routine dental examinations. Second, the low proportion of participants seeking professional care highlights the need for greater public awareness regarding available diagnostic and treatment options. Third, the absence of strong independent predictors reinforces the importance of comprehensive clinical evaluation that considers multiple contributing factors rather than focusing on a single presumed cause. Contemporary recommendations emphasize careful differential diagnosis, identification of predisposing factors, patient education, and individualized management strategies tailored to the underlying etiology of dentin exposure and symptom expression [8, 20, 29].

Finally, the findings should be interpreted in light of the study design. Because the assessment relied on self-reported symptoms without clinical confirmation, the results describe the epidemiology of self-reported dentin hypersensitivity rather than clinically diagnosed DH. Nevertheless, the study provides important baseline data from an underrepresented population and contributes to the limited evidence available on dentin hypersensitivity in Egypt and the broader Middle East and North Africa region. Future studies incorporating standardized clinical diagnostic protocols, objective assessment of gingival recession, dietary and erosive risk factors, and validated patient-reported outcome measures would provide a more comprehensive understanding of the determinants, patient burden, and long-term consequences of dentin hypersensitivity in this population [8, 25].

### Strengths and limitations

This study provides epidemiological data on self-reported dentin hypersensitivity (DH) among Egyptian adults, helping to address a gap in the epidemiological literature from Egypt and the broader Middle East and North Africa region. Strengths include the relatively large sample size, recruitment from multiple governorates, and the use of a structured questionnaire that underwent expert validation, pilot testing, and reliability assessment, demonstrating good internal consistency and temporal stability. In addition to estimating prevalence, the study evaluated symptom characteristics, triggering factors, oral hygiene behaviors, treatment-seeking patterns, and factors associated with symptom severity and self-reported gingival recession using multivariable regression analyses.

Several limitations should be considered. The cross-sectional design precludes causal inference, and all data were self-reported, introducing the possibility of recall bias, reporting bias, and misclassification. Importantly, DH was identified based on reported symptoms rather than clinical examination or standardized provocation testing; therefore, the findings reflect self-reported symptoms rather than clinically confirmed dentin hypersensitivity. Consequently, some participants may have reported symptoms not exclusively attributable to clinically defined DH or interpreted pain descriptors differently, which may explain the predominance of dull pain despite the classical description of DH as a short, sharp, stimulus-evoked pain. The use of convenience sampling from community and dental-care settings may limit the generalizability of the findings to the wider Egyptian population. Additionally, detailed symptom-related and behavioral information was collected only from participants reporting DH, preventing direct comparisons with unaffected individuals. Finally, although the 86 participants with self-reported DH provided sufficient statistical power for the planned analyses, the sample size may have limited the ability to detect weaker associations in multivariable models.

Despite these limitations, the study provides valuable baseline data from an underrepresented population and offers insights that may inform future epidemiological research, preventive strategies, and patient education initiatives related to dentin hypersensitivity.

## Conclusion

Self-reported dentin hypersensitivity affected nearly one-quarter of the surveyed Egyptian adults, with cold stimuli representing the most frequently reported trigger. Considerable variability was observed in symptom characteristics, oral hygiene practices, and self-reported gingival recession, highlighting the heterogeneous presentation of self-reported DH in this population. Although no significant independent predictors of severe symptoms or self-reported gingival recession were identified, the findings provide baseline epidemiological data on self-reported DH in an underrepresented population.

Future population-based studies incorporating standardized clinical diagnostic protocols, objective assessment of gingival recession, validated patient-reported outcome measures, and longitudinal follow-up are needed to confirm these findings and further clarify the determinants and impact of dentin hypersensitivity.

## Supplementary Information

Supplementary Material 1.

Supplementary Material 2.

## Data Availability

No datasets were generated or analysed during the current study.
