# Supplementary material for: Prevalence and oral hygiene–related correlates of self-reported dentin hypersensitivity among Egyptian adults: a cross-sectional study
Source: BMC Oral Health. 2026 Oct 5;26:1928. doi: 10.1186/s12903-026-09900-4 (PMC13640215; doi:10.1186/s12903-026-09900-4)
Supplement: Supplementary file 2 [file 12903_2026_9900_MOESM2_ESM.docx]

**Supplementary Material**

**Appendix S1. Dentin Hypersensitivity Questionnaire (DHQ)**

**Form Description:**
*This questionnaire aims to assess dentin hypersensitivity (DH) among adults. Participation is voluntary, and all responses will be kept confidential.*

**Section 1: Consent**

1. Do you agree to participate in this questionnaire?
   - Yes
   - No

**Section 2: Demographics**

2. Age:
   - 22–29 years
   - 30–39 years
   - 40–49 years
   - 50–59 years
   - 60 years and above
3. Gender:
   - Male
   - Female
   - Other / Prefer not to say
4. Place of birth (governorate):
   *(You may tick one or specify “Other”)*
   - Cairo
   - Alexandria
   - Giza
   - Sharqia
   - Qalyubia
   - Monufia
   - Dakahlia
   - Ismailia
   - Port Said
   - Suez
   - Minya
   - Luxor
   - Aswan
   - Matruh
   - Hurghada
   - Kafr el-Sheikh
   - New Valley
   - Red Sea
   - South Sinai
   - Beheira
   - Faiyum
   - Qena
   - Sohag
   - Asyut
   - Beni Suef
   - Damietta
   - 6th of October City
   - Gharbia
   - Helwan
   - Other: __________
5. Occupation: ___________________________

**Section 3: Lifestyle and Medical History**

6. Smoking status:
   - No
   - Yes, ≤ 20 cigarettes/day
   - Yes, ≥ 20 cigarettes/day
   - Other (e.g., occasional smoker, vaping): __________
7. Do you have any medical conditions? *(Select all that apply)*
   - Hypertension
   - Diabetes Mellitus
   - Cardiovascular disease
   - Thyroid disease
   - Asthma
   - Renal disease
   - Liver disease
   - No medical conditions
   - Other: __________

**Section 4: Dentin Hypersensitivity**

8. Do you currently experience dentin hypersensitivity?
   - Yes
   - No
9. If yes, what type of pain do you experience? *(Select all that apply)*
   - Sharp
   - Dull
   - Throbbing
10. Which stimuli trigger your DH? *(Select all that apply)*

- Hot
- Cold
- Sweet
- Sour
- Citrus fruits / juices
- Mouthwashes
- Toothbrush
- Chewing
- Other: __________

11. How often do you experience DH?

- Occasionally
- Often
- Constant / Persistent

12. Location of affected teeth: *(You may indicate multiple locations; a diagram can be provided for clarity)*

- Upper right jaw
- Lower right jaw
- Upper left jaw
- Lower left jaw
- Upper anteriors
- Lower anteriors

13.Have you previously sought treatment for DH?

- Yes
- No

**Section 5: Oral Hygiene Habits**

14.Bristle hardness of toothbrush used:

- Soft
- Medium
- Hard

15.Daily frequency of toothbrushing:

- None
- Once per day
- Twice per day
- Three or more times per day
- Other: __________

16.Do you brush with excessive force?

- Yes
- No

17.Toothbrushing technique:

- Horizontal
- Vertical
- Horizontal–vertical
- Circular
- Random / Other: __________

18. **Which type(s) of toothpaste do you regularly use? (Select all that apply)**

- Brand-specific desensitizing toothpaste (e.g., Sensodyne)
- Fluoride toothpaste (e.g., Colgate, Signal)
- Other desensitizing toothpaste (generic)
- Other: __________

**Section 6: Other Oral Health Factors**

19.Are you aware of bruxism or teeth grinding?

- Yes
- No

20. Do you experience gingival recession? *(Select all that apply)*

- No
- Upper jaw (specify teeth if possible)
- Lower jaw (specify teeth if possible)
- Upper anteriors
- Lower anteriors
